# Nationwide survey of formal mentorship characteristics and perceptions among resident physicians in Japan

**DOI:** 10.1186/s12909-026-09877-2

**Published:** 2026-07-04

**Authors:** Kohta Katayama, Yuji Nishizaki, Makoto Kikukawa, Toshihiko Takada, Kazuya Nagasaki, Taro Shimizu, Yu Yamamoto, Takashi Watari, Yasuharu Tokuda, Vineet Chopra, Yoshiyuki Ohira

**Affiliations:** 1 Department of General Internal Medicine, St. Marianna University School of Medicine, Kawasaki, Japan; 2 Division of Medical Education, Faculty of Medicine, Juntendo University, Tokyo, Japan; 3 Faculty of Medical Sciences Medical Education, School of Medicine, Kyusyu University, Fukuoka, Japan; 4 Department of General Medicine, Shirakawa Satellite for Teaching and Research (STAR), Fukushima Medical University, Shirakawa, Fukushima Japan; 5 Department of General and Family Medicine, Fujita Health University, Aichi, Japan; 6 Department of Diagnostic and Generalist Medicine, Dokkyo Medical University Hospital, Tochigi, Japan; 7 Division of General Medicine, Center for Community Medicine, Jichi Medical University, Tochigi, Japan; 8 Integrated Clinical Education Center, Kyoto University Hospital, Kyoto, Japan; 9 Muribushi Okinawa Center for Teaching Hospitals, Urasoe, Okinawa Japan; 10 Department of Medicine, University of Colorado School of Medicine, Aurora, USA

**Keywords:** Formal mentorship, Postgraduate medical education, Resident physicians, Gender diversity, Japan

## Abstract

**Background:**

Mentorship is considered essential in residency training, yet its structure and quality in Japan remain unclear.

**Methods:**

We conducted a nationwide, multi-center descriptive study of postgraduate year (PGY) 1–2 residents who completed both the General Medicine In-Training Examination (GM-ITE) and a formal mentorship survey in 2024. Residents who declined participation, provided no response to the survey, had incomplete survey responses, or did not have a formal mentor were excluded.

**Results:**

Of 9,179 residents who took the GM-ITE, 3,877 from 570 hospitals were analyzed. Most reported having one mentor (71.6%), and mentors were predominantly male (72.9%). Only 61.0% of programs offered lectures on mentorship. Overall, 46.1% of residents did not perceive feedback as specific or actionable, whereas 69.3% reported trusting their mentors. Mentoring interactions were relatively limited, with 60.4% of residents reporting meeting less than once a month.

**Conclusions:**

Among residents with formally assigned mentors, important gaps remained in mentor diversity, feedback quality, and mentoring engagement. Efforts to strengthen mentor training, improve feedback practices, and promote more structured mentoring interactions may help optimize residents’ educational and professional development.

**Supplementary Information:**

The online version contains supplementary material available at https://doi.org/10.1186/s12909-026-09877-2.

## Introduction

Mentorship is a dynamic, reciprocal process in which a more experienced professional, the mentor, supports the development of a less experienced colleague, referred to as the mentee, while also gaining insights and fulfillment from the relationship [1]. Rather than a one-sided transfer of knowledge, effective mentorship is built on mutual engagement, trust, and shared growth. In the context of medical education, mentorship has been associated with improved academic outcomes, enhanced psychosocial well-being, and greater satisfaction with clinical training [2]. Resident physicians in the early stages of postgraduate training often face demanding clinical responsibilities while simultaneously adjusting to new professional and social roles. This transitional period is accompanied by long working hours, uncertainty in clinical decision-making, and pressure to establish future career paths, all of which place them at increased risk of stress, depression, and burnout [3, 4]. Given this vulnerability, structured mentorship is considered a critical form of support, offering not only academic guidance but also psychosocial encouragement. Effective mentorship may therefore serve as a protective factor, helping residents cope with the challenges of residency and fostering resilience during this formative stage of their careers.

In Japan, mentorship has been increasingly recognized as an important component of residency training. Residency follows six years of medical school and consists of a two-year postgraduate program. National guidelines emphasize the need for supportive educational environments, including mentorship [5], yet the actual implementation of mentoring systems remains highly variable across institutions [6]. Previous studies have reported that many resident physicians have mentors and are generally satisfied with these relationships [6, 7]. However, other findings indicate that the presence of a mentor does not necessarily translate into measurable improvements in clinical competence, such as in-training examination performance [8]. Although some hospitals have introduced formal mentorship programs, comprehensive national-level data on how these systems are structured and experienced by residents are still lacking.

Building on this background, this study aims to provide a national overview of mentorship as assigned by residency programs in Japan. Specifically, we sought to describe the current status of formal mentorship by examining three domains: mentor characteristics (including number and gender), the perceived quality of mentorship (including the presence of lectures about mentorship, clarity of feedback, and trust in mentors), and the practical aspects of mentoring (including its frequency and duration). By addressing these dimensions, this study intends to highlight areas for improvement and contribute to the development of more effective mentorship systems to support resident physicians in Japan.

## Methods

### Study design and population

This was a nationwide, multi-center, descriptive study conducted in Japan to investigate the current status of mentorship among resident physicians. This study was conducted in accordance with the Japanese Ethical Guidelines for Medical and Health Research involving Human Subjects and adhered to the principles of the Declaration of Helsinki. The protocol was approved by the Ethics Review Board of the Japan Institute for Advancement of Medical Education Program (JAMEP) (24 − 15). Informed consent was obtained electronically from all participants before participation in the survey.

Eligible participants were postgraduate year (PGY)-1 and − 2 resident physicians training at teaching hospitals across Japan who completed both the General Medicine In-Training Examination (GM-ITE) and a self-reported clinical training environment survey administered immediately after the GM-ITE at the end of the 2024 academic year. Residents who declined participation, provided no response to the survey, reported not having a formal mentor, had missing responses to mentorship questions, or had missing responses to clinical training environment questions were excluded. We collected information on resident and institutional characteristics, including hospital type, location, number of beds, year of residency, gender, number of inpatients under their care, and duty hours per week.

The GM-ITE is a nationwide computer-based examination developed by the JAMEP that assesses core clinical competencies among resident physicians in Japan [9–11]. In 2023, 9,580 resident physicians from 696 hospitals participated in the examination [11]. Because the GM-ITE is administered across a broad range of residency programs and teaching hospitals throughout Japan, it provided a practical platform for conducting a large-scale nationwide survey of mentorship experiences among resident physicians.

### Data collection on mentorship

We developed the survey instrument for this study. The questionnaire was developed through an iterative process involving a review of the mentorship literature and discussions among investigators with expertise in postgraduate medical education. Two items assessing feedback quality and trust in mentors were adapted from the Mentoring Meeting Assessment Tool (MMAT) [12]. In the survey, we defined mentorship as a reciprocal relationship in which an advanced-career mentor supports the growth of the mentee while also developing professionally through interaction. We focused on formal mentorship assigned by residency programs and collected information in three domains: mentor characteristics (including number and gender), the perceived quality of mentorship (including the presence of lectures about mentorship, clarity of feedback, and trust in mentors), and the practical aspects of mentoring (including its frequency and duration).

Participants selected the number of formally assigned mentors from 11 response options (0 to 10 or more). For analysis, these responses were categorized into three groups: one mentor, two mentors, and three or more mentors. Mentor gender was reported as male, female, or both.

Questions on clarity of feedback and trust in mentors were adapted from the Mentoring Meeting Assessment Tool (MMAT) [12]. Mentoring frequency was reported using 11 response options (0 to 10 or more times per month) and was categorized for analysis as less than once per month (including no meetings), once per month, and two or more times per month. The duration of mentoring sessions was reported using 11 response options ranging from none to 91 min or more and was categorized into three groups: no mentoring sessions, 10–30 min, and 40 min or longer. The full English version of the questionnaire is provided in the Supplementary File.

### Statistical analyses

We summarized the participants’ characteristics. Frequencies and percentages were calculated for dichotomous and ordered categorical variables, respectively. Descriptive statistics were also used to characterize the survey results. All analyses were conducted using Stata/SE software, version 15 (Stata Corp. College Station, TX, USA).

## Results

**Fig. 1:**
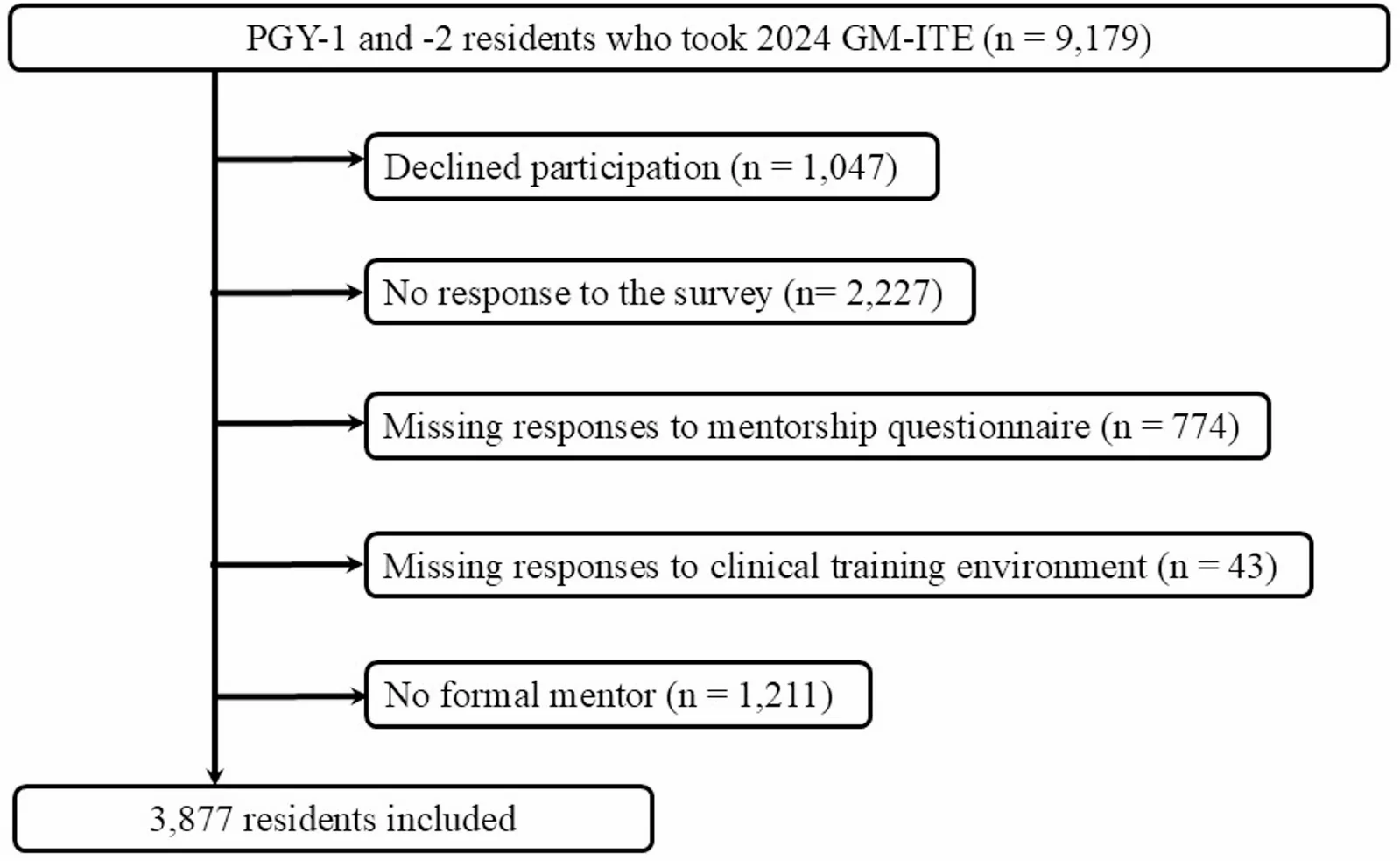
Flow diagram of study participants

Figure 1 shows the flow of participant selection. Of the 9,179 resident physicians who took the GM-ITE in 2024, residents who declined participation, provided no response to the survey, had incomplete survey responses, or did not have a formal mentor were excluded. Ultimately, 3,877 residents from 570 hospitals were included in the analysis. Participant characteristics are presented in Table 1.

**Table 1:** Characteristics of Resident Physicians Included in the Study

	All (*N* = 3,877)
Hospital characteristics	
Hospital types (%)	
University	485 (12.5)
University branch	307 (7.9)
Community	3,085 (79.6)
Located urban area (%)	
Urban	1,318 (34.0)
Rural	2,559 (66.0)
Number of beds (%)	
1 to 299	304 (7.8)
300 to 599	2,103 (54.2)
600 to 899	990 (25.5)
900 or more	480 (12.3)
Resident characteristics	
Gender (%)	
Men	2,604 (67.2)
Women	1,273 (32.8)
PGY (%)	
PGY-1	2,023 (52.2)
PGY-2	1,854 (47.8)
Number of inpatients under their care (%)	
0–4	1,482 (38.2)
5–9	1,813 (46.8)
10–14	312 (8.1)
15 or more	96 (2.5)
Unknown	174 (4.5)
Duty hour per week (%)	
50 or less	1,710 (44.1)
50–59	1,424 (36.7)
60–69	559 (14.4)
70–79	98 (2.5)
80 or more	86 (2.2)

Data are presented as frequency (%). PGY, postgraduate year

### Mentor characteristics

**Fig. 2:**
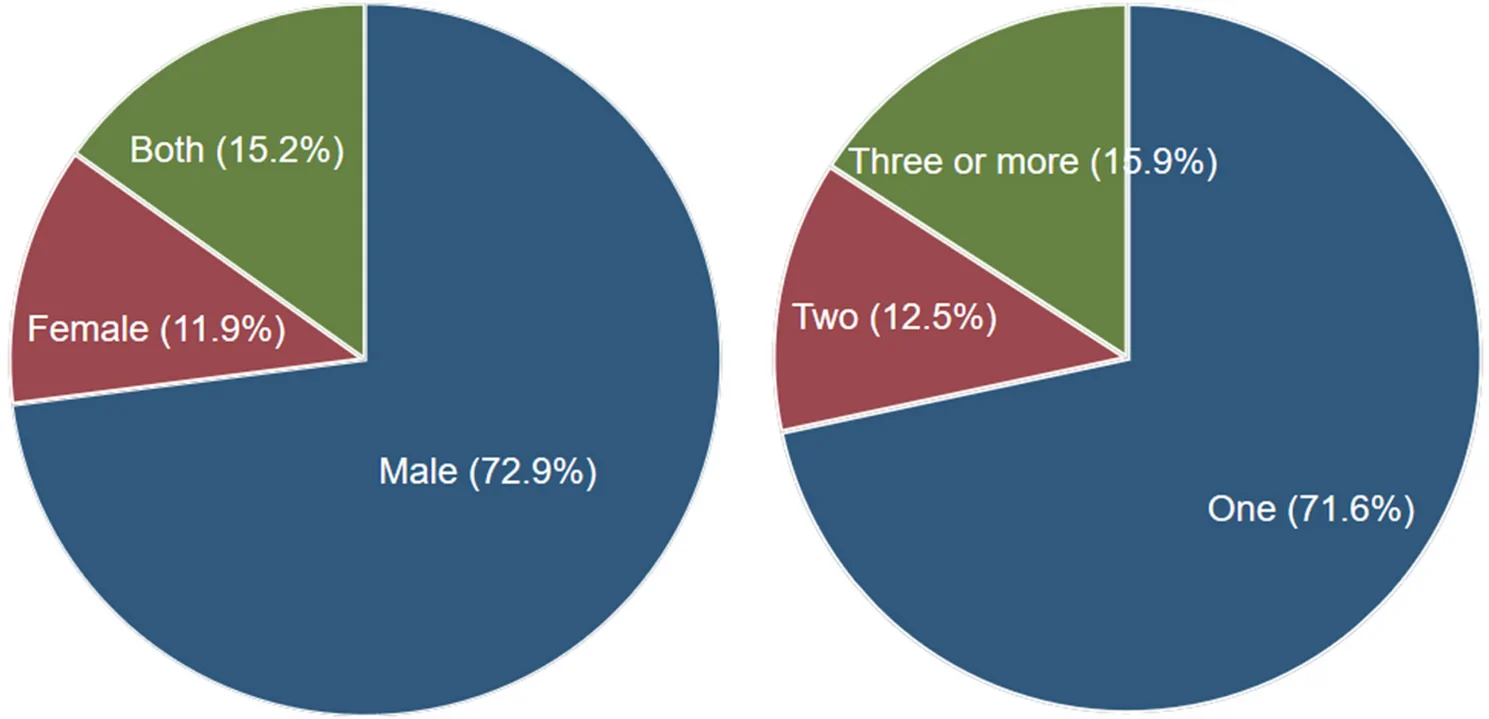
Mentor characteristics: mentor gender (left) and number of mentors (right)

Mentor characteristics are summarized in Fig. 2. Most residents (71.6%) reported having a single assigned mentor, whereas 12.5% reported having two mentors, and 15.9% had three or more mentors. Regarding mentor gender, the majority were male (72.9%), followed by both male and female mentors (15.2%) and female mentors (11.9%).

### Quality of mentorship

Overall, 61.0% of residency programs provided lectures on mentorship. Residents’ perceptions of feedback quality and trust in their mentors varied (Table 2). While 53.9% of residents agreed or strongly agreed that “feedback was specific, actionable, and focused on how to improve,” 46.1% did not (mean [SD] score, 2.5 [0.9] on a 5-point scale). In contrast, 69.3% agreed or strongly agreed that they trusted their mentors’ commitment to their success, yielding a higher mean score of 2.8 (SD 0.9).

**Table 2:** Residents’ perceptions of the quality of mentorship

	Strongly Disagree *n* (%)	Disagree *n* (%)	Neither agree nor disagree *n* (%)	Agree *n* (%)	Strongly agree *n* (%)	Mean (SD)
Feedback was specific, actionable, and focused on how to improve	144 (3.7)	252 (6.5)	1,392 (35.9)	1,680 (43.3)	409 (10.6)	2.5* (0.90)
Trust that mentors are committed to my success	85 (2.2)	138 (3.6)	968 (25.0)	1,935 (49.9)	751 (19.4)	2.8* (0.86)

*Mean scores from 1 (strongly disagree) to 5 (strongly agree) with SDs are presented

### Practical features of mentorship

**Fig. 3:**
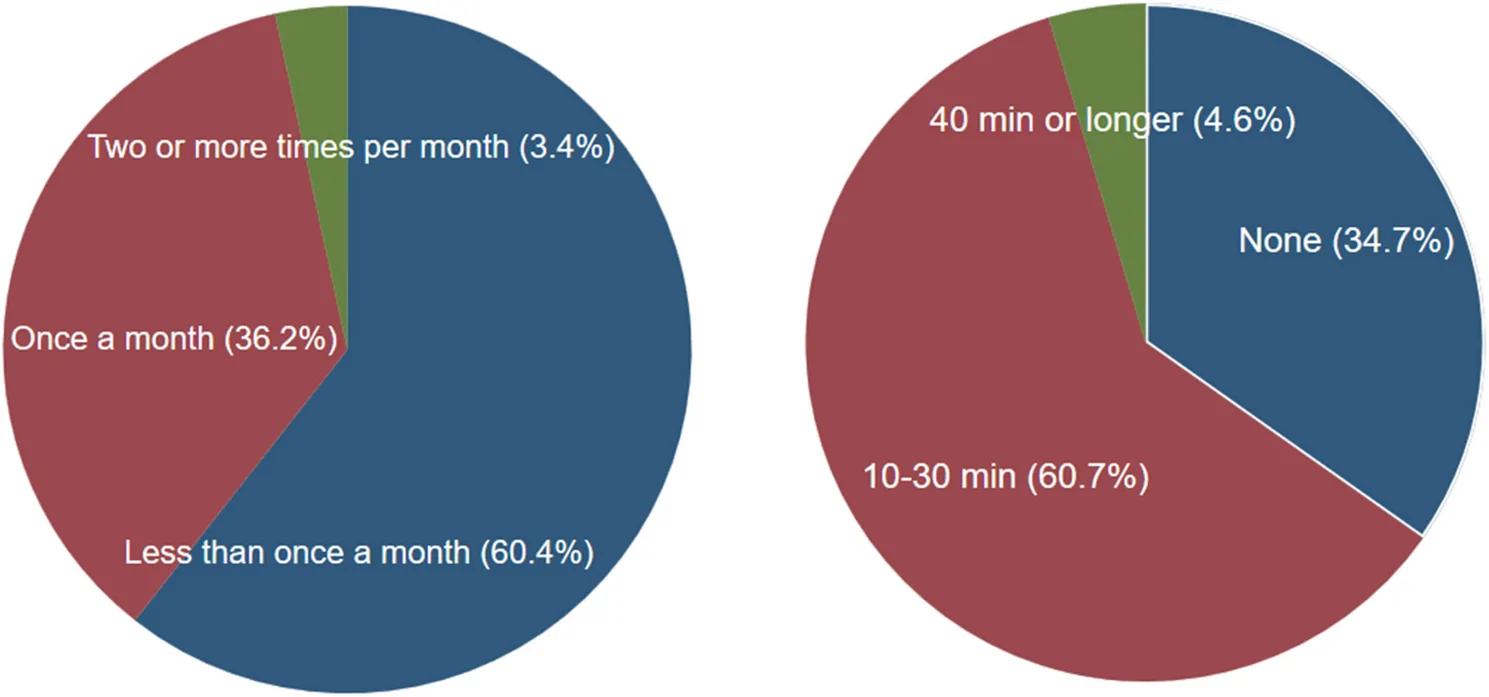
Practical features of mentorship: frequency of mentoring sessions (left) and duration (right)

With respect to the frequency of mentoring sessions (Fig. 3), only 3.4% of residents met their mentors two or more times per month, while 36.2% reported once per month, and 60.4% reported less than once per month (including no meetings). Regarding duration, 34.7% reported having no mentoring sessions, 60.7% reported 10–30 min, and only 4.6% reported 40 min or longer.

## Discussion

In this nationwide descriptive study of resident physicians in Japan, 71.6% of residents reported having a single formal mentor, whereas only 27.1% reported having at least one female mentor. Even among residents with formally assigned mentors, 34.7% reported no mentoring meetings, and 60.7% reported mentoring sessions lasting 30 min or less. Although 61.0% of residency programs offered lectures on mentorship, only 53.9% of residents agreed or strongly agreed that their mentors provided clear and actionable feedback, whereas 69.3% agreed or strongly agreed that they trusted their mentors. To our knowledge, this is the first nationwide survey to comprehensively describe the structural and experiential aspects of assigned mentorship among Japanese resident physicians, highlighting important gaps in both the availability and quality of mentoring.

One notable finding of this study was that only 27.1% of residents reported having at least one female mentor. Although this proportion is comparable to the overall proportion of female physicians in Japan (24.4%) [13], opportunities for residents to engage with female mentors remain limited. This imbalance may reflect the broader gender disparity in the Japanese medical workforce. Although the number of female physicians in Japan has been increasing in recent years, their representation in leadership and supervisory positions remains considerably lower than that of their male counterparts [14]. The scarcity of female mentors suggests that female resident physicians may lack accessible role models who can provide concrete career pathways. Female resident physicians may particularly benefit from female mentors who are able to share experiences and advice on challenges specific to women, such as balancing career advancement with major life events or addressing gender bias in the workplace [15]. Without such support, they may face disadvantages in their career development. Furthermore, female physicians are more likely to experience additional psychological burdens arising from gender-based expectations in both professional and personal domains [15]. Given the protective role of mentorship in resident physicians’ mental health [6], strengthening mentorship opportunities for female resident physicians should be a priority. The growing proportion of female physicians in Japan suggests that the number of female mentors will likely increase in the future, helping promote greater gender diversity in residency programs [14].

Another important observation was the generally low quality of mentorship reported by Japanese resident physicians. Although more than two-thirds of residents expressed trust in their mentors, nearly half did not perceive the feedback they received as specific or actionable. In contrast to a U.S. study where the average rating of feedback quality on a five-point Likert scale was as high as 4.7, the mean score in our study was only 2.5 [12]. Several factors may account for this discrepancy, including cultural background, the overall quality of mentorship, and generational differences in communication styles. Japanese mentoring relationships have been described as strongly hierarchical and paternalistic [16, 17]. According to Hofstede’s cultural dimensions, Japan has a moderately high power distance compared with many Western countries, meaning that hierarchical relationships and respect for authority are more strongly embedded in educational and workplace interactions, but simultaneously inhibit mentees from questioning or requesting clarification when feedback is vague [18]. Notably, Japanese clinical residency guidelines as well as the model core curriculum for medical education lack specific descriptions of the behaviors or practices that constitute successful mentorship [5, 19]. Faculty development should therefore emphasize training mentors to deliver constructive feedback, structuring mentoring sessions, and fostering both professional growth and psychosocial support. In addition, our findings showed that mentoring sessions were infrequent, with 60.4% of resident physicians meeting their mentors less than once a month (including no meetings). Generational differences may contribute to the observed gap. Such limited opportunities for interaction may be particularly problematic given the expectations of millennial resident physicians, who often value clear instructions, rapid responses, and frequent short meetings to exchange information [20]. This mismatch between the traditional style of mentors and the preferences of millennial mentees may partly explain why residents perceive the overall quality of mentorship to be low.

Several limitations warrant consideration. First, selection bias is possible. Our sampling frame comprised resident physicians who both sat for the GM-ITE and completed the post-exam survey. Because program directors decide whether residents take the GM-ITE, our sample may over-represent hospitals with stronger educational infrastructure and support for assessment. Second, all data were self-reported by resident physicians; we had no parallel information from mentors or objective program metrics, which may tilt interpretations toward the mentees’ perspective. Third, the descriptive cross-sectional design precludes causal inference and does not allow us to estimate the effect of mentorship on outcomes such as clinical performance or well-being. We also did not systematically capture potential downsides of mentoring (e.g., mismatch, over-dependence, or conflict), and our exclusion of residents without a mentor limits generalizability to settings where mentorship is absent or purely informal. Fourth, the mentorship questionnaire was developed specifically for this study and was not formally pilot tested or psychometrically validated, which may have limited the validity of some mentorship-related measures. Fifth, we also did not examine specialty-specific differences in mentorship characteristics or satisfaction with mentorship. Because residents could select multiple specialties and the interpretation of specialty preferences differed between PGY-1 and PGY-2 residents, such analyses were beyond the scope of the present study. Nevertheless, residents pursuing different specialties may have distinct educational and mentorship needs. A previous study has suggested that personal and leadership-related characteristics vary across specialty preferences among Japanese residents, which may influence mentorship expectations [21]. Future studies should therefore investigate specialty-specific mentorship needs and optimal mentoring approaches. Finally, measurement and reporting biases cannot be ruled out, since responses relied on residents’ recall and perceptions. However, the potential for social desirability bias was mitigated by the fact that program directors were not informed of either the questionnaire responses or GM-ITE scores, which may have encouraged more candid reporting.

In conclusion, this nationwide study provides the first comprehensive description of formal mentorship among resident physicians in Japan. Among residents with formally assigned mentors, important gaps remained in mentor diversity, feedback quality, and the frequency of mentoring interactions. These findings suggest opportunities to strengthen mentorship programs through enhanced mentor development, improved feedback practices, and more structured mentoring engagement. Future studies should explore how mentorship can be tailored to residents’ diverse educational needs and evaluate its impact on educational and professional outcomes.

## Supplementary Information

Supplementary Material 1.

## Data Availability

Residents who joined in this study did not give consent for their data to be shared publicly, so supporting data is not available. The corresponding author will respond to inquiries on the data analyses in this study.

## Abbreviations

GM-ITE: General Medicine In-Training Examination
JAMEP: Japan Organization of Advancing Medical Education Program
MMAT: Mentoring Meeting Assessment Tool
PGME: postgraduate medical education
PGY: postgraduate year
