# Supplementary material for: Nationwide survey of formal mentorship characteristics and perceptions among resident physicians in Japan
Source: BMC Med Educ. 2026 Jul 4;26:1536. doi: 10.1186/s12909-026-09877-2 (PMC13617937; doi:10.1186/s12909-026-09877-2)
Supplement: Supplementary file 1 [file 12909_2026_9877_MOESM1_ESM.docx]

**Supplementary File: English Version of the Mentorship Survey**

**In this survey, mentorship was defined as a reciprocal relationship in which an advanced-career mentor supports the growth of the mentee while also developing professionally through interaction.**

**1. How many mentors have been formally assigned to you by your residency program or hospital?**
□ 0　□ 1　□ 2　□ 3　□ 4　□ 5　□ 6　□ 7　□ 8　□ 9　□ 10 or more

**2. What is the gender of the mentor(s) formally assigned to you?**
**(If you have more than one mentor, please select all that apply.)**
□ Male　□ Female

**3. Does your residency program or hospital provide lectures or educational sessions about mentorship?**
□ Yes　□ No

**4. The feedback provided by my formally assigned mentor was clear, actionable, and focused on how to improve.**
□ Strongly disagree　□ Disagree　□ Neither agree nor disagree　□ Agree　□ Strongly agree

**5. I trust that my formally assigned mentor is committed to supporting my success in residency training.**
□ Strongly disagree　□ Disagree　□ Neither agree nor disagree　□ Agree　□ Strongly agree

**6. How many mentoring meetings do you have with your formally assigned mentor per month?**
□ 0　□ 1　□ 2　□ 3　□ 4　□ 5　□ 6　□ 7　□ 8　□ 9　□ 10 or more

**7. Approximately how long does each mentoring meeting last?**
□ None　□ 10 minutes　□ 20 minutes　□ 30 minutes　□ 40 minutes　□ 50 minutes　□ 60 minutes　□ 70 minutes　□ 80 minutes　□ 90 minutes　□ 91 minutes or more
